# Attitude of primiparous women towards their preference for delivery method: a qualitative content analysis

**DOI:** 10.1186/s13690-019-0364-y

**Published:** 2019-08-20

**Authors:** Alireza Khatony, Ali Soroush, Bahare Andayeshgar, Neda Saedpanah, Alireza Abdi

**Affiliations:** 1 Health Institute, Social Development and Health Promotion Research Center, Kermanshah University of Medical Sciences, Kermanshah, Iran; 2 Clinical Research Development Center of Imam Reza Hospital, Kermanshah University of Medical Sciences, Kermanshah, Iran; 3 Students Research Committee, School of Nursing and Midwifery, Kermanshah University of Medical Sciences, Kermanshah, Iran; 4 Nursing Department, School of Nursing and Midwifery, Dowlat Abad, Kermanshah, Iran

**Keywords:** Attitude, Parity, Women, Delivery, Qualitative study

## Abstract

**Background:**

The delivery method is one of the primary concerns of primiparous women. Today, due to the fear of delivery, the number of cesarean sections has increased significantly. Considering the importance of choosing the type of delivery and the lack of study in this regard, the present study was conducted to explain the attitude of primiparous women towards their preference for delivery method.

**Method:**

In this qualitative study, in-depth interview was conducted with 12 primiparous women. The transcripts of the interviews were read carefully by the researcher for several times. Finally, using the analytical method, the codes were extracted and subsequent subcategories and main categories were determined. To assess the credibility of the data, the Denzin & Lincoln’s criteria were used, which include Credibility, Transferability, Dependability, and Confirmability.

**Results:**

Because of inexperience in delivery and ambiguity in process, most of the woman had a specific fear and they commonly affected by other advices and experiences. The majority of the participants had desire to vaginal delivery with announcing its advantages such as shortage of pain process, easy breast feeding, and mother ability to better carryout the newborn works. Other participants believed on adverse effects of normal vaginal delivery including bladder prolapse, uterus rupture, and probability of experience of both happenings (CS and NVD), frequent examination, pain and urine incontinence. Some stated cesarean section have benefits of less injury to neonate and preserving her/his beauty.

**Conclusion:**

Results showed that, choosing a delivery method is difficult for primiparous women, and their attitude toward the type of delivery is influenced by subjective and internal factors. Giving awareness to primiparous women greatly enhances their tendency towards vaginal delivery.

## Background

Childbirth is one of the divine blessings for the reproduction of mankind on earth, which has been continuously taken place since the birth of Adam [1]. Although, childbirth brings happiness to many women, it is a unique event that is associated with severe pain. Majority of women describe labor pain as the most severe pain experienced [2]. Caesarean Section (CS) has become the most common surgery in many parts of the world [3]. Many women wish to have CS without a medical indication. About 10–14% of CSs in the world are done with the request of the mothers without medical indication [4]. Today, due to the fear of childbirth, the number of CS cases has increased substantially [5]. Evidence suggests that multiparous women prefer CS, and primiparous women prefer vaginal delivery [6]. Today, the rate of CS without medical indication has increased dramatically. In one study in 137 countries, the rate of CS in 54 countries was below 10%, in 41 countries was between 10 and 15% and in 69 countries was above 15%. Meanwhile, the rate of CS in Iran (41.9%) is ranked second after Brazil (45.9%), which is higher than the World Health Organization’s acceptable level [7]. The results of a systematic review and meta-analysis study in Iran (2014) estimated the rate of CS about 48% [8].

The psychological factors affecting the choice of delivery method include; education, economic strength, anxiety, and self-confidence [9]. Based on the available evidence, the most important factors affecting the choice of delivery method among Iranian primiparous women include fear, incorrect information broadcasting methods, social factors, and the opinions of physicians and healthcare workers [10–13]. The focus of these studies has been on external factors such as decision-making by physicians and healthcare personnel. On the other hand, there are subjective and internal factors that affect the choice of delivery method. Knowledge of pregnant women about childbirth comes from social exchanges and others’ experiences. Evidence emphasizes that benefiting from the experiences of others is an effective way to help pregnant women choose the type of delivery [14]. Given the fact that optional CS imposes high costs on the healthcare system and leads to many complications for mothers, recognizing the subjective and internal factors affecting their preference for delivery method helps us to educate the primiparous mothers and reduce their fear and anxiety caused by vaginal delivery and also encourage them to choose natural delivery method. Regarding the high rate of CS in Iran, and Importance of doing studies in this issue, the researchers decided to study the attitude of primiparous women towards their preference for delivery method during this qualitative study.

## Methods

### Type of study

The present qualitative study was conducted in 2017 with the aim of explaining the views of primiparous women on the type of delivery. Data collection lasted about three month from June to august 2017. This qualitative study was done as content analysis. Qualitative studies are based on the naturalistic paradigm, in which the uniqueness of phenome is supported [15]. The findings of a qualitative study are consequences of human experiences, and indeed it is emerged from interaction between researcher and participants.

### Participants

The study population consisted of all primiparous women referred to Imam Reza Hospital in Kermanshah-West of Iran. The participants were purposefully selected. For data collection, the researcher referred to obstetrics clinic and pregnancy classes of the hospital, then the purposes of the study were explained clearly to the woman, they assured about anonymity and confidentiality of the personal information. The woman that consented to participate in the study, invited to come to the researcher office (one of the hospital room) and a semi-structured interview was conducted with them via some open questions. The participants were between month of fifth-eighth of pregnancy and those did not desire to be interviewed of discontinue the interview were excluded from the study.

The number of participants was determined based on the data saturation. It is notable, the researcher tried to have no pre-assumption about the research content and the interviews was done by third researcher who is female and married and has an experience of normal vaginal delivery.

### Data collection method

The data were collected by the semi-structured interview. The guide questions were: “What is your priority for your delivery? What are the advantages and disadvantages of vaginal delivery and CS from your point of view? What are the factors affecting the choice of delivery from your point of view”? Also to clarify concepts, words such as “why, how, and please explain more” were used. After obtaining permission from the Ethics Committee of Kermanshah University of Medical Sciences (KUMS), the samples were selected purposefully and interviews were started after explaining the aims of study and obtaining written informed consent from the samples. Each interview was recorded by audio recorder and soon after, the text of the interview was carefully written and then typed. The transcripts were repeatedly read by the researcher and compared with the recorded voice. This action was carried out to facilitate further control over the data and data analysis.

### Data analysis

After the first interview, data analysis was started and immediately after each interview, the text of the interview entered into the software Maxqda and analyzed using qualitative content analysis. In a content analysis approach, the researcher gains a deep understanding of the concepts involved in the subject, and words and sentences are categorized into main categories and subcategories that make the phenomenon easier to understand [16]. For this purpose, the transcripts of the interviews were read carefully by the researcher, and after gaining a general understanding of them, similar codes were given to the concepts and sentences that had the same meaning. Finally, according to the extracted codes, the main categories and subcategories were determined. Interviews continued until data saturation that occurs when the new code is not extracted and the members of the research team have verified the codes to ensure the accuracy of the data saturation. The data saturation was achieved in the ten interviews, however we conducted to another interviews to confident of saturation.

### Trustworthiness

The four criteria that Denzin & Lincoln have proposed to evaluate the descriptive research include credibility, transferability, dependability, and confirmability [17]. In this study, in order to increase the credibility of the data, in-depth and long interviews were conducted and repeated questions were used to resolve the uncertainty and to ensure the participants’ response. Finally, the texts of the interviews were reviewed by other researchers. One of the limitations of qualitative studies is the transferability of their results. Nevertheless, for the purpose of results’ transferability, the phenomenon was carefully studied and the characteristics of the age and education of the participants were explained. Furthermore, the results of the study were given to three primiparous women who had not been participating in the study and their experiences were compared with the results of the current study, resulting in the approval of the codes and categories by them. Dependability is a sign of honesty in research, and shows the data have been properly collected and not influenced by the researcher’s view. To ensure this, all stages of the study were expressed to be readily judged by readers. Confirmability means the relevance of the results to the goal of the study. In order to achieve this, the recorded interviews, their transcripts and codes were given to other research team members who were qualified in the field of qualitative research and confirmed the accuracy of coding.

### Ethics considerations

The Ethics Committee of KUMS approved the study with the code: ir.kums.rec.1396.18. The objectives of the study were explained to the participants and emphasis was placed on the confidentiality of their detail and information. Written and informed consent was obtained from all participants.

## Results

In this study, twelve pregnant women aged 18–32 years old entered the study. The criteria for entering the study included being pregnant for the first time and women who had a pregnancy experience in the past were excluded (Table 1). After analyzing the qualitative content of the interviews, 280 codes were extracted. The findings consisted of three main categories and nine subcategories. The main categories included; “Access to information resources”, “Attitude towards delivery” and “Fear”. Nine subcategories were also resulted from the data analysis, including: “Formal information resource”, “Informal information resource”, “Advantages of vaginal delivery”, “Advantages of caesarean section”, “Disadvantages of vaginal delivery”, “Disadvantages of caesarean section”, “Fear of harming the infant”, “Fear of pain intolerance”, and “Fear of personnel’s behavior” (Table 2).

**Table 1:** Participants’ characteristics

Participants	Age(Yrs.)	Grade
First	27	Diploma
Second	32	MSc.
Third	21	Diploma
Fourth	23	Diploma
Fifth	23	Diploma
Sixth	19	Under diploma
seventh	27	BSc.
Eights	25	BSc.
Ninth	24	Diploma
Tenth	30	Diploma
Eleventh	27	Diploma
twelfth	18	Under diploma

**Table 2:** Extracted categories and sub-categories’ attitude of primiparous women towards the choice of delivery method

categories	sub-categories
Access to information resources	Formal resources
	Informal resources
Attitude towards delivery	Advantages of virginal delivery
	Advantages of Caesarean Section
	Disadvantages of virginal delivery
	Disadvantages of Caesarean Section
Fear	Fear of pain intolerance
	Fear of harming the infant
	Fear of personnel’s behavior

### Access to information resources

The views and opinions of most participants had been shaped by the information obtained from formal and informal resources. Formal resources included books, mass media and educational programs, and informal resources included the experience and advice of others and watching online film.

#### Formal resources

Some participants referred to media and educational programs as sources of information. The priority these people for delivery was vaginal delivery. One of the participants in this regard stated: “The media, especially TV, with their programs, documentaries and photos are very influential on the minds and decision of the people in the community” (First interview, a 27 years old woman). Another participant who had taken part in pregnancy training said: “I participated in natural birth preparation classes because I had heard those who had participated in these classes have been having a very easy delivery, I mean, they have delivered their baby as soon as they arrived at the hospital with minimum discomfort” (Fifth interview, a 23 years old woman). Another participant in this regard stated: “Television and all media recommend natural delivery and this is effective” (Fourth interview, a 23 years old woman).

#### Informal resources

All participants had gained much of their information from the words and experiences of others. One of the pregnant women in this regard stated: “My mother had a vaginal delivery and my aunt had a CS. My mother says, vaginal delivery is better for the baby, and has less postpartum problems compared to the cesarean section. She has had 4 vaginal deliveries and believes that vaginal delivery is better. But my aunt had lots of difficulty after her cesarean section. She could not handle breastfeeding, because she had a lot of pain due her abdomen surgical site. She says she cannot lift a heavy objects to this day due to her CS”(Ninth interview, a 24 years old woman). Another participant stated: “A few days ago, my aunt delivered her baby at our home and the baby was born very easy. I did not even hear my aunt’s screaming at all. It was a good experience for me” (Twelfth Interview, an 18 years old woman). Another participant referred to the impact of watching movie on the attitude toward delivery: “I have already watched a film on vaginal delivery, but in the movie, it is easier than what I have heard from others” (Sixth interview, a 19 years old woman).

### Attitude towards delivery

Most participants had a tendency towards vaginal delivery and pointed to the shorter duration of pain, easier breastfeeding, and the ability of mother to carry out child-related activities as the advantages of vaginal delivery. Also, from the viewpoint of the participants, the disadvantages of vaginal delivery included complications such as prolapsed bladder and rupture of the uterus, the probability of experiencing both vaginal delivery and cesarean section, frequent medical examinations, pain and incontinence. Some participants believed that cesarean section has some advantages, such as posing less harm to the baby and maintaining beauty compared to vaginal delivery. Also the biggest disadvantage of cesarean section from the viewpoint of most participants were the risks and side effects associated with this type of delivery. Medical errors, the possibility of adhesion and bleeding, the probability of post-cesarean infection, loss of blood, rupture of various layers of the abdomen and sutures, and possible side-effects of anesthesia were some of the disadvantages pointed out by the participants. This category included four sub-categories; advantages of vaginal delivery, advantages of caesarean section, disadvantages of vaginal delivery, and disadvantages of caesarean section.

#### Advantages of vaginal delivery

Most participants pointed to the ability of mothers after vaginal delivery and easier lactation as the most obvious advantages of vaginal delivery. In this regard, one of the participants in the eleventh interview stated: “The mother, after a vaginal delivery, will be up and walking after a day but CS keeps mothers in hospital for a few days for care and attention that may be required even at home. However, after vaginal delivery, you are much better and can take care of yourself”(Eleventh interview, a 27 years old woman).

In regard to easier lactation, one participant said: “Everyone says that in vaginal delivery, you can look after your baby better, get better more quickly and breastfeed the baby. I have seen that, in CS the mother has so much pain that she cannot breastfeed the baby and do anything for the baby, but after a vaginal delivery, the pain is less and the mother can be more comfortable and able to take care of the baby and breastfeed him”(Ninth interview, a 24 years old woman). In the fourth and fifth interviews, this issue was confirmed by the sentences such as: “After a vaginal delivery, you can take care of the baby and breastfeed him, but you need care and rest after cesarean section” and also “You will be comfortable as soon as the baby is born, and you can communicate with the baby, cuddle him and breastfeed him, but in the caesarean section you may not have milk for at least three days”.

#### Advantages of CS

Some participants were interested in cesarean section as they were afraid that natural delivery may harm their infants. They pointed to the lack of harm to their babies as one of the advantages of cesarean section. One participant in this regard stated: “The problems that occur to the infant during vaginal delivery do not occur during cesarean section. I have seen that during vaginal delivery, the pelvis or/and the shoulder of the baby was dislocated, and the neck of a baby was tilted. I have seen all these and according to the experience of others, I like to have a caesarean section”(Tenth interview, a 30 years old woman).

Maintaining the beauty was also important for some women and they considered it as an advantage of cesarean section. One participant in this regard stated: “In the cesarean section, the appearance of the genital system will not change, but this is not the case in vaginal delivery. This is important for some, although nowadays, many people do cosmetic surgeries” (Twelfth interview, an 18 years old woman). Another participant acknowledged this issue with the following statement: “Vaginal delivery causes the form of pelvic and genitalia to change, but this is not the case in the cesarean section, and the original form of the genitalia will not change. This is important for some men as they do not like the form of women’s genitalia to change, my husband wants me to have caesarean section.” (Eleventh interview, a 27 years old woman).

#### Disadvantages of vaginal delivery

From some participants’ point of view, the disadvantages of vaginal delivery included complications such as bladder prolapsed and uterine rupture, probability of experiencing both vaginal and caesarian deliveries, frequent medical examinations, pain and incontinence. One participant regarding the experience of both deliveries stated: “Once, I saw a woman endured a lot of pain during vaginal delivery and tried to have normal birth as long as she could, but eventually she was sent to the operating room as an emergency. This is awful because she was in pain both before and after the delivery; these cases sometimes happen”(Second interview, a 32 years old woman). Another participants in regard to the disadvantages of vaginal delivery stated: “Each of the two vaginal delivery and cesarean section has its own complications and disadvantages. There is a chance of bladder prolapse, uterine rupture and repeated examinations in vaginal delivery. Also, vaginal delivery is not hygienic” (First interview, a 27 years old woman). In the tenth interview, this issue was acknowledged by the following sentence: “My sister, after 15 years of her vaginal delivery, still has pain and bladder prolapsed, so when she goes to toilet, she has problem. During vaginal delivery, due to the pressure on the pelvis, the pelvis will not remain the same”. Another participant stated: “One of my concerns for vaginal delivery is that, I have heard they constantly perform medical examination and since most hospitals are teaching hospitals, if any midwife or student come and insert her hand inside me, I worry this may cause illness or infection as their hands might have been dirty. Another thing that I don’t like about vaginal delivery is that, it poses lots of pressure on the intestine that may cause incontinence. I’m so scared of that, and do not like it to happen to me” (Fifth interview, a 23 years old woman).

#### Disadvantages of CS

Medical errors, the possibility of adhesion and bleeding, the probability of infection after CS, loss of blood, tearing of different layers of the abdomen and sutures, and side effects of anesthesia were some of the disadvantages of CS mentioned by some participants. One participant in this regard stated: “In the cesarean section, since the uterus is opened, the chance of infection is greater, there is a lot of bleeding and there may be complications for women. There will also be stitches and scares on the abdomen……. For cesarean section, an anesthetic ampoule is injected into the spinal cord that some people say have side effects such as lower back pain”(Fifth interview, a 23 years old woman). Also, in the twelfth interview, another participant with the following phrases referred to the disadvantages of cesarean section: “Caesarean section has its own complications, such as lower back pain, headache and leg pain. Also, after cesarean section, the abdomen remains enlarged for longer whereas in vaginal delivery, the uterus returns to its normal size quicker and the abdomen becomes smaller”. Another participant in regard to the medical errors stated: “Cesarean section may be dangerous for the mother as it may harm the bowel or other organs, or even a surgical instrument may be left behind in the abdomen” (Tenth interviewer, a 30 years old woman). Another participant in the eleventh interview confirmed this by saying: “Sometimes a mistake is done during cesarean section, such as leaving something behind in the mother’s abdomen, or making mistakes that cause complications or do not completely evacuating the blood. But this is not the case in vaginal delivery and the blood is completely evacuated and medical errors are less.”

### Fear

Fear was one of the main categories, which was mentioned by all participants. In primiparous women, lack of previous experience of delivery and lack of knowledge about this process lead to the fear of delivery. In this regard, most participants were worried about the health of their infants and the effect of delivery method on it. They were also worried about medical errors, intolerance to pain and consequent inability to perform childbirth, personnel’s attitude, and complications of anesthetic drugs used in cesarean section. This category had three sub-categories, including “fear of pain intolerance”, “fear of harming the infant” and “fear of personnel’s behavior”.

#### Fear of pain intolerance

Most participants were afraid of labor pain, and this feeling was caused by the words and experiences of others. One participant in this regard said: “I do not tolerate pain at all and I feel that I am going to die, which is indescribable. I’ve seen women who go to the labor room and experience a great deal of pain. But if you are sedated, you will not feel pain.”(Seventh interview, a 27 years old woman). Another participant in this regard stated: “I am scared of vaginal delivery and I cannot stand it. I have heard many things about the pain of vaginal delivery from others. I have even heard that some people wait for a vaginal delivery in a delivery room for several days, and at the end, they are forced to have cesarean section. I think it’s hard to experience the pain of both deliveries”(Sixth interview, a 19 years old woman).

#### Fear of harming the infant

Some participants were worried about the harming of their baby during the childbirth. It seemed that, the nature of vaginal delivery and hearing the negative experiences of others have led to this fear. One of the participants stated: “What scares me the most is that, they may drop the baby during vaginal delivery and harm his head, or apply lots of pressure on the baby’s head and change its shape. These things really scare me. We had a neighbor whose vaginal delivery seriously damaged her baby’s hand.”(Eighth interview, a 25 years old woman). Another participant stated: “I am more concerned about my baby than myself, and I do not want my baby to be harmed. If my water bag is teared, my pelvis is tight or the baby is turned, my baby may get injured or suffer from mental retardation” (Second interview, a 32 years old woman).

#### Fear of personnel’s behavior

Due to the need for support during labor and the impossibility of spouse presence in the delivery room, some participants were afraid of personnel’s behavior. Hearing others’ experiences or encountering personnel’s inappropriate behavior during previous visits may be involved in creating such feeling in the participants. One of the participants in this regard stated: “I would love to see nurses or doctors who are caring for me have a good behavior with each other. I’m so scared that they may shout at me and that makes me very anxious. If my mother and my husband stay with me, I will not be afraid of anything” (Eighth interview, a 25 years old woman).

Another participant in this regard said: “Personnel’s ethics have an effect on childbirth. In some hospitals, those who work in the delivery room are very tedious and this creates anxiety, and if there is a sign or symptom, you cannot tell them or easily communicate with them. But, if they are good-tempered and well-behaved, you can explain everything to them to help you have easier delivery”(Third interview, a 21 years old woman).

## Discussion

The purpose of this study was to explain the attitude of primiparous women towards their preference for delivery method. One of the main categories was access to information resources. In our study, the participants consisted of primiparous women with the first gestational experience who were obtaining their information from various sources, including formal and informal sources. Books, mass media and educational programs as formal sources and experiences and words of others in relation to childbirth were recognized as an informal source of information.

Evidence suggests that pregnant women gain their information from a variety of resources, such as the experiences of others [18, 19], and mass media [19, 20]. The results of other studies indicate the effect of educational programs on the choice of delivery method and the reduction of CS [21, 22]. In our opinion, the pregnant woman need more information regarding vaginal delivery. Their attendance in group educational classes encourage them to VD, because group discussion and meeting the woman with the same condition may reduce fear. Some evidences confirm the effects of group care before delivery, and the woman share their positive experience each other and take appropriate information from the leaders [23, 24], also some approaches such as deep breathing and yoga exercises have been suggested in this regard [25].

In our study too, some participants also pointed to the impact of educational programs on the priority of vaginal delivery.

Research evidence suggests that primiparous women are hesitant in choosing the type of delivery, and are easily influenced by the words of people close to them, such as mothers and spouses [19, 26, 27]. Our results are in line with these studies. One of the ways to encourage the primiparous women to choose vaginal delivery is to use formal information resources such as mass media.

The attitude toward delivery was another category that contained the participants’ understanding of benefits and disadvantages of CS and vaginal delivery. Most participants referred to the shorter duration of pain, easier breastfeeding, and the ability of mother to carry out child-related activities as the advantages of vaginal delivery. In this regard, pregnant Canadian mothers believed that, vaginal delivery has advantages such as better baby’s health, easier breastfeeding and faster recovery [28]. In our study, some participants considered pain and incontinence as the disadvantages of CS. In this regard, the results of a study in North America showed that vaginal delivery is dirty, incompatible, and humiliating from the view point of some women, and urinary incontinence is among its complications [29].

From the participants’ point of view, maintaining the beauty was among the benefits of CS. Evidence also suggests that, one of the main benefits of CS is maintaining the beauty of genital area [29, 30]. Another benefit of CS according to the participants was lesser possibility of harming the infant, while evidence suggests that babies born by CS method are more at risk of some diseases [31–34]. Most participants were familiar with some of the complications of CS, such as the possibility of bleeding and infection, and problems with lactation. Other studies also indicate that complications of CS are more than vaginal delivery, which include bleeding and lactation problems [33, 35]. In our view, informing pregnant women about the complications of caesarean section can greatly reduce the number of CSs that lack medical indication.

Another main category that explained the attitudes of primiparous women was the “fear”. Most participants had doubts about their preference for delivery, and the fear of vaginal delivery was a feeling that primiparous women had perceived even before the childbirth. Other studies also suggest that fear of childbirth is a feeling that some women experience [5, 36–38].

One of the subcategories of fear was the fear of pain intolerance. Pain as a natural but inevitable part of childbirth leads to mothers’ fear and anxiety. Evidence suggests that women who intend to have CS are more likely to be afraid of labor pain [39–43]. It seems using different approaches to reduce pain during delivery such as warm shower, rhythmic breathing and educating about labor could decrease the fear of vaginal delivery, which demands more studies. Participating in care courses before delivery and educational classes as well as doing regular exercise are other suggested methods [44].

The fear of harming the infant during childbirth was another subcategory of the fear, which was mentioned by most participants. They believed that, this is more likely to occur in vaginal delivery and the baby’s shoulder, hand or foot may get harmed during the childbirth. Evidence suggests that fear of harming the infant is one of the reasons for being scared of the childbirth [39, 45]. The risk of neonate damage is potential in both methods of deliveries, and it is 2% about vaginal delivery and 1.1% in cesarean [46]. The reasons of delivery fear in Iranian woman is related to hospital environment and low quality of cares [47], in which the roles of midwiferies are more vital. A qualitative study results showed, the midwiferies have low motivation to engage in care of patients with vaginal delivery by two reasons of lack of responsibility and low salary [48]. In our opinion, good communication of physicians and midwifes with pregnant woman and take well assurance to them reduce their stress and maybe incline them to do vaginal delivery.

Fear of inappropriate behavior of personnel was another subcategory of fear. During pregnancy, pregnant mothers need emotional support, and in this regard, personnel can reduce their fear and anxiety by having appropriate behavior towards them. It is obvious that, inappropriate behavior of maternity ward staffs may exacerbate the fear and concern of mothers. The results of a study showed that, not trusting the personnel of maternity ward was one of the reasons for the fear of pregnant women [39]. Evidence has shown that personnel are an important decision-maker for mothers [40, 43] and support of obstetric personnel affects the delivery process and facilitates a good delivery [49, 50]. As some participants indicated, we believe, the attendance of husbands in delivery room could reduce pain and anxiety during vaginal delivery, however, we have such guideline (attendance of husband in delivery room) in Kermanshah hospitals but it do not implemented, by reasons of some facilities and cultural restraints.

### Limitations

There was some limitations in the study, one of them was related to physical and emotional disorders of the participants, and we tried to provide a comfort situation for them and encouraging the pregnant woman to participate in the study by offering some information about the importance of the issue. Another limitation is lack of generalizability of the results, which is the nature of qualitative research.

## Conclusion

It is difficult to choose a method of delivery for primiparous women, and their attitude towards the type of delivery is influenced by subjective and internal factors. By increasing the awareness of primiparous women about the benefits of vaginal delivery and risks of caesarean section, we can greatly increase the tendency of primiparous women towards vaginal delivery and reduce the rate of Cesarean Sections that have low medical indication.

## Data Availability

Data available by contacting the corresponding author.

## Abbreviation

CS: Cesarean section
